# (*E*)-1-[(2-Amino-5-nitro­phen­yl)iminio­meth­yl]naphthalen-2-olate

**DOI:** 10.1107/S1600536810014923

**Published:** 2010-04-30

**Authors:** Abeer Mohamed Farag, Siang Guan Teoh, Hasnah Osman, Suchada Chantrapromma, Hoong-Kun Fun

**Affiliations:** a School of Chemical Sciences, Universiti Sains Malaysia, 11800 USM, Penang, Malaysia; b Crystal Materials Research Unit, Department of Chemistry, Faculty of Science, Prince of Songkla University, Hat-Yai, Songkhla 90112, Thailand; c X-ray Crystallography Unit, School of Physics, Universiti Sains Malaysia, 11800 USM, Penang, Malaysia

## Abstract

The title Schiff base compound, C_17_H_13_N_3_O_3_, crystallizes in a zwitterionic form and exists in a *trans* configuration about the C=N bond. The mol­ecule is slightly twisted, the dihedral angle between the benzene ring and naphthalene ring system being 10.80 (9)°. The nitro group is twisted relative to the plane of the benzene ring [dihedral angle = 8.88 (12)°]. Bifurcated intra­molecular N—H⋯N and N—H⋯O hydrogen bonds formed between iminium groups and amine N atoms and naphthalen-2-olate O atoms generate *S*(5) and *S*(6) ring motifs, respectively. In the crystal, neighbouring zwitterions are linked through weak C—H⋯O inter­actions, giving rise to screw chains along [010]. Mol­ecules in these chains are linked to those of an adjacent chains through N—H⋯O hydrogen bonds and weak C—H⋯O inter­actions, forming sheets parallel to the *ac* plane. O⋯C [2.895 (3) Å] short contacts and π–π inter­actions [centroid–centroid distance = 3.8249 (19) Å] are also observed.

## Related literature

For bond-length data, see: Allen *et al.* (1987 ▶). For hydrogen-bond motifs, see: Bernstein *et al.* (1995 ▶). For background to Schiff bases and their applications, see: Eltayeb *et al.* (2007 ▶; 2008 ▶); Dao *et al.* (2000 ▶); Kagkelari *et al.* (2009 ▶); Karthikeyan *et al.* (2006 ▶); Sondhi *et al.* (2006 ▶); Sriram *et al.* (2006 ▶). For related structures, see: Eltayeb *et al.* (2009 ▶; 2010 ▶). For the stability of the temperature controller used in the data collection, see Cosier & Glazer, (1986 ▶).
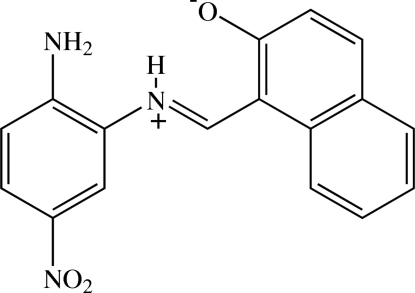


## Experimental

### 

#### Crystal data

C_17_H_13_N_3_O_3_
*M* *_r_* = 307.30
Monoclinic, 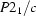

*a* = 10.369 (4) Å
*b* = 4.6442 (18) Å
*c* = 28.539 (9) Å
β = 103.548 (12)°
*V* = 1336.1 (8) Å^3^
*Z* = 4
Mo *K*α radiation
μ = 0.11 mm^−1^
*T* = 100 K
0.48 × 0.10 × 0.04 mm

#### Data collection

Bruker APEXII DUO CCD area-detector diffractometer
Absorption correction: multi-scan (*SADABS*; Bruker, 2009 ▶) *T*_min_ = 0.950, *T*_max_ = 0.996
14018 measured reflections
3887 independent reflections
2341 reflections with *I* > 2σ(*I*)
*R*_int_ = 0.057

#### Refinement

*R*[*F* ^2^ > 2σ(*F* ^2^)] = 0.057
*wR*(*F* ^2^) = 0.151
*S* = 1.02
3887 reflections
220 parameters
H atoms treated by a mixture of independent and constrained refinement
Δρ_max_ = 0.36 e Å^−3^
Δρ_min_ = −0.30 e Å^−3^

### 

Data collection: *APEX2* (Bruker, 2009 ▶); cell refinement: *SAINT* (Bruker, 2009 ▶); data reduction: *SAINT*; program(s) used to solve structure: *SHELXTL* (Sheldrick, 2008 ▶); program(s) used to refine structure: *SHELXTL*; molecular graphics: *SHELXTL*; software used to prepare material for publication: *SHELXTL* and *PLATON* (Spek, 2009 ▶).

## Supplementary Material

Crystal structure: contains datablocks global, I. DOI: 10.1107/S1600536810014923/sj2769sup1.cif

Structure factors: contains datablocks I. DOI: 10.1107/S1600536810014923/sj2769Isup2.hkl

Additional supplementary materials:  crystallographic information; 3D view; checkCIF report

## Figures and Tables

**Table 1:** Hydrogen-bond geometry (Å, °)

*D*—H⋯*A*	*D*—H	H⋯*A*	*D*⋯*A*	*D*—H⋯*A*
N1—H1*N*1⋯O1	1.01 (3)	1.61 (3)	2.505 (2)	146 (3)
N1—H1*N*1⋯N2	1.01 (3)	2.39 (3)	2.737 (3)	100 (2)
N2—H1*N*2⋯O1^i^	0.89 (3)	2.47 (3)	3.219 (3)	142.0 (19)
N2—H2*N*2⋯O1^ii^	0.95 (3)	1.98 (3)	2.879 (3)	158.6 (19)
C6—H6*A*⋯O3^iii^	0.93	2.57	3.489 (3)	168
C15—H15*A*⋯O2^iv^	0.93	2.51	3.161 (3)	127

Symmetry codes: (i) 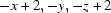; (ii) 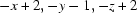; (iii) 
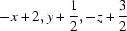
; (iv) 
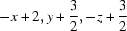
.

**Fig. 1.**
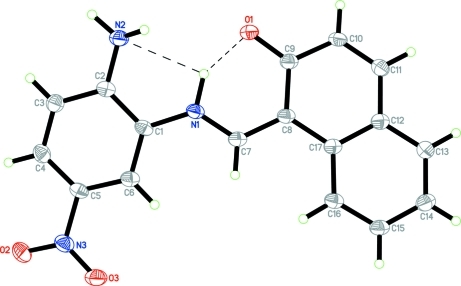
The molecular structure of the title compound, with 50° probability displacement ellipsoids and the atom-numbering scheme. Intramolecular hydrogen bonds are shown as dashed lines." is correct.

**Fig. 2.**
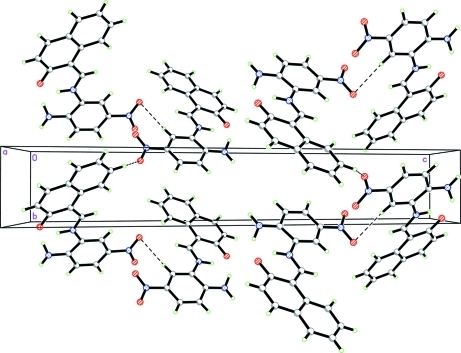
The crystal packing of the title compound viewed down the a, showing screw chains running along the b axis. Hydrogen bonds are shown as dashed lines.

**Fig. 3.**
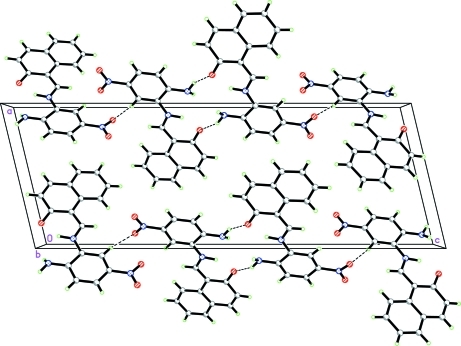
The crystal packing of the title compound viewed down the b, showing sheet parallel to the ac plane. Hydrogen bonds are shown as dashed lines.

## supplementary crystallographic information

### Comment

Schiff base ligands are members of an important class of compounds, possessing a wide spectrum of biological and pharmacological activities such as antibacterial and antifungal (Karthikeyan *et al.*, 2006), anticancer (Dao *et al.*, 2000), anti-HIV (Sriram *et al.*, 2006), activities. Apart of these activities they have also been used as ligands to study coordination chemistry (Kagkelari *et al.*, 2009). As part of our ongoing research on the synthesis of Schiff base ligands and their complexes (Eltayeb *et al.*, 2007; 2008; 2009; 2010), the title compound (I) was synthesized and its crystal structure was determined. The title Schiff base ligand in neutral form was tested for anti-inflammatory, analgesic and kinase inhibition activities and showed moderate anti-inflammatory and analgesic activities (Sondhi *et al.*, 2006).

The molecule of (I) (Fig. 1), C_13_H_9_BrNO_2_, crystallizes in a zwitterionic form with cationic iminium and anionic enolate, and exists in a *trans* configuration about the C═N bond [1.315 (3) Å] and the torsion angle C1–N1–C7–C8 = 175.18 (19)°. The naphthalene ring system [C8–C17] is planar with the *r.m.s.* 0.0069 (2) Å. The molecule is twisted with the dihedral angle between the benzene and naphthalene rings being 10.80 (9)°. The nitro group is twisted relative to the plane of the C8–C13 benzene ring with an interplanar angle of 8.88 (12)° and torsion angles O2–N3–C5–C4 = 8.4 (3) and O3–N3–C5–C4 = -171.70 (19)°.  Bifurcated intramolecular N1—H1N1···N2 and N1—H1N1···O1 hydrogen bonds (Fig.1) which formed between the NH^+^ and amino N atom and to the naphthalen-2-olate O^-^ generates an S(5) and S(6) ring motifs, respectively (Bernstein *et al.*, 1995). The bond distances are in normal ranges (Allen *et al.*, 1987) and comparable with the related structures (Eltayeb *et al.*, 2009; 2010).

In the crystal packing, neighbouring zwitterions are linked through weak C—H···O interactions (Table 1) giving rise to screw chains along the [010] direction (Fig. 2). Molecules in a chain are linked to those of adjacent chains through N—H···O(naphthalen-2-olate) hydrogen bonds and weak C—H···O(nitro) interactions (Table 1, Fig. 3), forming sheets parallel to the *ac* plane. O···C [2.895 (3) Å] short contacts and π–π interactions with centroid···centroid distances of 3.8249 (19) Å are also observed.

### Experimental

The title compound was synthesized by adding 2-hydroxy-1-naphthaldehyde (0.688 g, 4 mmol) to the solution of 4-nitrobenzene-1,2-diamine (0.306 g, 2 mmol) in ethanol (30 ml). The mixture was refluxed with stirring for 3 hrs. The resultant solid was obtained and then filtered and washed with ethanol. Red plate-shaped single crystals of the title compound suitable for *x*-ray structure determination were obtained from acetone by slow evaporation at room temperature after several days.

### Refinement

Amine and iminium H atoms were located from the difference maps and were refined isotropically. The remaining H atoms were placed in calculated positions with d(C—H) = 0.93 Å for aromatic and CH atoms and the *U*_iso_ values were constrained to be 1.2*U*_eq_ of the carrier atoms. The highest residual electron density peak is located at 0.70 Å from H4A and the deepest hole is located at 0.65 Å from C4.

### Crystal data

**Table:**

C_17_H_13_N_3_O_3_	*F*(000) = 640
*M**_r_* = 307.30	*D*_x_ = 1.528 Mg m^−^^3^
Monoclinic, *P*2_1_/*c*	Mo *K*α radiation, λ = 0.71073 Å
Hall symbol: -P 2ybc	Cell parameters from 3887 reflections
*a* = 10.369 (4) Å	θ = 1.5–30.0°
*b* = 4.6442 (18) Å	µ = 0.11 mm^−^^1^
*c* = 28.539 (9) Å	*T* = 100 K
β = 103.548 (12)°	Plate, red
*V* = 1336.1 (8) Å^3^	0.48 × 0.10 × 0.04 mm
*Z* = 4	

### Data collection

**Table:**

Bruker APEXII DUO CCD area-detector diffractometer	3887 independent reflections
Radiation source: sealed tube	2341 reflections with *I* > 2σ(*I*)
graphite	*R*_int_ = 0.057
φ and ω scans	θ_max_ = 30.0°, θ_min_ = 1.5°
Absorption correction: multi-scan (*SADABS*; Bruker, 2009)	*h* = −14→13
*T*_min_ = 0.950, *T*_max_ = 0.996	*k* = −6→6
14018 measured reflections	*l* = −39→40

### Refinement

**Table:**

Refinement on *F*^2^	Primary atom site location: structure-invariant direct methods
Least-squares matrix: full	Secondary atom site location: difference Fourier map
*R*[*F*^2^ > 2σ(*F*^2^)] = 0.057	Hydrogen site location: inferred from neighbouring sites
*wR*(*F*^2^) = 0.151	H atoms treated by a mixture of independent and constrained refinement
*S* = 1.01	*w* = 1/[σ^2^(*F*_o_^2^) + (0.059*P*)^2^ + 0.6347*P*] where *P* = (*F*_o_^2^ + 2*F*_c_^2^)/3
3887 reflections	(Δ/σ)_max_ = 0.001
220 parameters	Δρ_max_ = 0.36 e Å^−^^3^
0 restraints	Δρ_min_ = −0.30 e Å^−^^3^

### Special details

**Table:**

Experimental. The crystal was placed in the cold stream of an Oxford Cryosystems Cobra open-flow nitrogen cryostat (Cosier & Glazer, 1986) operating at 100.0 (1) K.
Geometry. All esds (except the esd in the dihedral angle between two l.s. planes) are estimated using the full covariance matrix. The cell esds are taken into account individually in the estimation of esds in distances, angles and torsion angles; correlations between esds in cell parameters are only used when they are defined by crystal symmetry. An approximate (isotropic) treatment of cell esds is used for estimating esds involving l.s. planes.
Refinement. Refinement of F^2^ against ALL reflections. The weighted R-factor wR and goodness of fit S are based on F^2^, conventional R-factors R are based on F, with F set to zero for negative F^2^. The threshold expression of F^2^ > 2sigma(F^2^) is used only for calculating R-factors(gt) etc. and is not relevant to the choice of reflections for refinement. R-factors based on F^2^ are statistically about twice as large as those based on F, and R- factors based on ALL data will be even larger.

### Fractional atomic coordinates and isotropic or equivalent isotropic displacement parameters (Å^2^)

**Table:**

	*x*	*y*	*z*	*U*_iso_*/*U*_eq_	
O1	0.82332 (14)	−0.0589 (3)	0.96873 (5)	0.0254 (4)	
O2	1.22099 (15)	−0.6304 (4)	0.76236 (5)	0.0338 (4)	
O3	1.09865 (15)	−0.2495 (4)	0.74798 (5)	0.0317 (4)	
N1	0.92876 (16)	−0.1176 (4)	0.89879 (5)	0.0198 (4)	
H1N1	0.911 (3)	−0.151 (6)	0.9315 (10)	0.053 (8)*	
N2	1.08296 (18)	−0.4892 (5)	0.96268 (6)	0.0250 (4)	
H1N2	1.068 (2)	−0.318 (6)	0.9746 (9)	0.038 (7)*	
H2N2	1.134 (2)	−0.630 (6)	0.9828 (9)	0.036 (7)*	
N3	1.15214 (17)	−0.4461 (4)	0.77485 (6)	0.0254 (4)	
C1	1.02284 (18)	−0.2851 (5)	0.88271 (6)	0.0189 (4)	
C2	1.09971 (19)	−0.4753 (5)	0.91675 (6)	0.0200 (4)	
C3	1.1889 (2)	−0.6596 (5)	0.90188 (7)	0.0231 (5)	
H3A	1.2383	−0.7889	0.9238	0.028*	
C4	1.2048 (2)	−0.6533 (5)	0.85552 (7)	0.0234 (5)	
H4A	1.2636	−0.7783	0.8458	0.028*	
C5	1.13185 (19)	−0.4580 (5)	0.82357 (6)	0.0215 (4)	
C6	1.04003 (19)	−0.2757 (5)	0.83600 (6)	0.0216 (4)	
H6A	0.9907	−0.1492	0.8135	0.026*	
C7	0.84656 (19)	0.0765 (5)	0.87499 (6)	0.0196 (4)	
H7A	0.8514	0.1269	0.8439	0.023*	
C8	0.75161 (19)	0.2092 (5)	0.89575 (6)	0.0194 (4)	
C9	0.7424 (2)	0.1231 (5)	0.94325 (6)	0.0211 (4)	
C10	0.6390 (2)	0.2452 (5)	0.96265 (6)	0.0243 (5)	
H10A	0.6300	0.1872	0.9929	0.029*	
C11	0.5547 (2)	0.4420 (5)	0.93792 (7)	0.0242 (5)	
H11A	0.4894	0.5173	0.9518	0.029*	
C12	0.56177 (19)	0.5396 (5)	0.89118 (6)	0.0208 (4)	
C13	0.4720 (2)	0.7454 (5)	0.86662 (7)	0.0239 (5)	
H13A	0.4088	0.8222	0.8814	0.029*	
C14	0.4757 (2)	0.8357 (5)	0.82110 (7)	0.0252 (5)	
H14A	0.4148	0.9701	0.8049	0.030*	
C15	0.5722 (2)	0.7223 (5)	0.79976 (6)	0.0230 (5)	
H15A	0.5758	0.7834	0.7691	0.028*	
C16	0.66212 (19)	0.5223 (5)	0.82295 (6)	0.0214 (4)	
H16A	0.7258	0.4515	0.8078	0.026*	
C17	0.65970 (19)	0.4222 (5)	0.86945 (6)	0.0188 (4)	

### Atomic displacement parameters (Å^2^)

**Table:**

	*U*^11^	*U*^22^	*U*^33^	*U*^12^	*U*^13^	*U*^23^
O1	0.0339 (8)	0.0264 (9)	0.0168 (6)	0.0063 (7)	0.0075 (6)	0.0041 (6)
O2	0.0391 (9)	0.0402 (11)	0.0257 (7)	0.0115 (8)	0.0148 (7)	−0.0053 (7)
O3	0.0429 (9)	0.0331 (10)	0.0218 (7)	0.0053 (8)	0.0127 (6)	0.0051 (7)
N1	0.0263 (8)	0.0187 (10)	0.0156 (7)	−0.0005 (7)	0.0069 (6)	−0.0004 (7)
N2	0.0335 (10)	0.0246 (11)	0.0176 (8)	0.0047 (9)	0.0075 (7)	0.0008 (8)
N3	0.0306 (9)	0.0270 (11)	0.0204 (8)	0.0009 (8)	0.0097 (7)	−0.0028 (7)
C1	0.0249 (9)	0.0159 (11)	0.0177 (8)	−0.0038 (8)	0.0085 (7)	−0.0033 (7)
C2	0.0245 (9)	0.0185 (12)	0.0177 (8)	−0.0038 (9)	0.0063 (7)	−0.0012 (8)
C3	0.0257 (10)	0.0203 (12)	0.0237 (9)	0.0012 (9)	0.0065 (8)	0.0008 (8)
C4	0.0260 (10)	0.0207 (12)	0.0249 (9)	0.0014 (9)	0.0086 (8)	−0.0032 (8)
C5	0.0250 (9)	0.0247 (13)	0.0160 (8)	−0.0022 (9)	0.0075 (7)	−0.0033 (8)
C6	0.0268 (9)	0.0208 (12)	0.0173 (8)	−0.0022 (9)	0.0053 (7)	−0.0005 (8)
C7	0.0262 (9)	0.0171 (11)	0.0155 (8)	−0.0028 (8)	0.0049 (7)	−0.0001 (7)
C8	0.0250 (9)	0.0178 (11)	0.0152 (8)	−0.0017 (8)	0.0046 (7)	−0.0003 (8)
C9	0.0284 (10)	0.0182 (11)	0.0165 (8)	−0.0003 (9)	0.0045 (7)	0.0000 (8)
C10	0.0343 (11)	0.0246 (13)	0.0158 (8)	0.0029 (10)	0.0098 (8)	0.0029 (8)
C11	0.0289 (10)	0.0241 (13)	0.0211 (9)	0.0016 (9)	0.0093 (8)	−0.0007 (8)
C12	0.0249 (9)	0.0190 (12)	0.0182 (8)	−0.0031 (9)	0.0047 (7)	−0.0005 (8)
C13	0.0270 (10)	0.0233 (13)	0.0218 (9)	0.0025 (9)	0.0063 (8)	0.0000 (8)
C14	0.0300 (10)	0.0233 (13)	0.0212 (9)	−0.0001 (9)	0.0035 (8)	0.0021 (8)
C15	0.0299 (10)	0.0232 (12)	0.0155 (8)	−0.0023 (9)	0.0046 (7)	0.0008 (8)
C16	0.0272 (10)	0.0205 (12)	0.0173 (8)	−0.0035 (9)	0.0069 (7)	−0.0011 (8)
C17	0.0238 (9)	0.0162 (11)	0.0160 (8)	−0.0039 (8)	0.0039 (7)	−0.0008 (7)

### Geometric parameters (Å, °)

**Table:**

O1—C9	1.289 (2)	C7—C8	1.405 (3)
O2—N3	1.220 (2)	C7—H7A	0.9300
O3—N3	1.237 (2)	C8—C9	1.438 (3)
N1—C7	1.315 (3)	C8—C17	1.454 (3)
N1—C1	1.406 (2)	C9—C10	1.434 (3)
N1—H1N1	1.00 (3)	C10—C11	1.344 (3)
N2—C2	1.364 (2)	C10—H10A	0.9300
N2—H1N2	0.89 (3)	C11—C12	1.427 (3)
N2—H2N2	0.94 (3)	C11—H11A	0.9300
N3—C5	1.455 (2)	C12—C13	1.402 (3)
C1—C6	1.386 (2)	C12—C17	1.417 (3)
C1—C2	1.413 (3)	C13—C14	1.374 (3)
C2—C3	1.397 (3)	C13—H13A	0.9300
C3—C4	1.371 (3)	C14—C15	1.391 (3)
C3—H3A	0.9300	C14—H14A	0.9300
C4—C5	1.380 (3)	C15—C16	1.372 (3)
C4—H4A	0.9300	C15—H15A	0.9300
C5—C6	1.381 (3)	C16—C17	1.412 (3)
C6—H6A	0.9300	C16—H16A	0.9300
			
C7—N1—C1	128.76 (16)	C7—C8—C9	118.92 (18)
C7—N1—H1N1	110.5 (16)	C7—C8—C17	121.33 (16)
C1—N1—H1N1	120.5 (16)	C9—C8—C17	119.71 (17)
C2—N2—H1N2	113.4 (17)	O1—C9—C10	119.21 (17)
C2—N2—H2N2	116.1 (15)	O1—C9—C8	122.43 (18)
H1N2—N2—H2N2	120 (2)	C10—C9—C8	118.36 (18)
O2—N3—O3	123.00 (17)	C11—C10—C9	121.25 (17)
O2—N3—C5	118.54 (18)	C11—C10—H10A	119.4
O3—N3—C5	118.46 (17)	C9—C10—H10A	119.4
C6—C1—N1	123.48 (18)	C10—C11—C12	122.52 (19)
C6—C1—C2	120.14 (18)	C10—C11—H11A	118.7
N1—C1—C2	116.36 (16)	C12—C11—H11A	118.7
N2—C2—C3	120.35 (19)	C13—C12—C17	120.24 (17)
N2—C2—C1	120.83 (19)	C13—C12—C11	120.69 (18)
C3—C2—C1	118.74 (17)	C17—C12—C11	119.05 (18)
C4—C3—C2	121.19 (19)	C14—C13—C12	121.21 (19)
C4—C3—H3A	119.4	C14—C13—H13A	119.4
C2—C3—H3A	119.4	C12—C13—H13A	119.4
C3—C4—C5	118.71 (19)	C13—C14—C15	118.7 (2)
C3—C4—H4A	120.6	C13—C14—H14A	120.6
C5—C4—H4A	120.6	C15—C14—H14A	120.6
C4—C5—C6	122.52 (17)	C16—C15—C14	121.52 (18)
C4—C5—N3	118.49 (18)	C16—C15—H15A	119.2
C6—C5—N3	118.99 (18)	C14—C15—H15A	119.2
C5—C6—C1	118.63 (19)	C15—C16—C17	121.11 (19)
C5—C6—H6A	120.7	C15—C16—H16A	119.4
C1—C6—H6A	120.7	C17—C16—H16A	119.4
N1—C7—C8	121.09 (17)	C16—C17—C12	117.18 (18)
N1—C7—H7A	119.5	C16—C17—C8	123.74 (18)
C8—C7—H7A	119.5	C12—C17—C8	119.08 (16)
			
C7—N1—C1—C6	−2.5 (3)	C17—C8—C9—O1	−178.14 (19)
C7—N1—C1—C2	179.1 (2)	C7—C8—C9—C10	−175.65 (19)
C6—C1—C2—N2	−179.3 (2)	C17—C8—C9—C10	2.0 (3)
N1—C1—C2—N2	−0.8 (3)	O1—C9—C10—C11	178.1 (2)
C6—C1—C2—C3	−2.4 (3)	C8—C9—C10—C11	−2.0 (3)
N1—C1—C2—C3	176.07 (18)	C9—C10—C11—C12	0.5 (3)
N2—C2—C3—C4	178.5 (2)	C10—C11—C12—C13	−179.9 (2)
C1—C2—C3—C4	1.6 (3)	C10—C11—C12—C17	1.1 (3)
C2—C3—C4—C5	0.8 (3)	C17—C12—C13—C14	0.6 (3)
C3—C4—C5—C6	−2.4 (3)	C11—C12—C13—C14	−178.3 (2)
C3—C4—C5—N3	178.11 (19)	C12—C13—C14—C15	−1.0 (3)
O2—N3—C5—C4	8.4 (3)	C13—C14—C15—C16	0.5 (3)
O3—N3—C5—C4	−171.70 (19)	C14—C15—C16—C17	0.4 (3)
O2—N3—C5—C6	−171.2 (2)	C15—C16—C17—C12	−0.8 (3)
O3—N3—C5—C6	8.8 (3)	C15—C16—C17—C8	179.5 (2)
C4—C5—C6—C1	1.5 (3)	C13—C12—C17—C16	0.3 (3)
N3—C5—C6—C1	−178.97 (19)	C11—C12—C17—C16	179.27 (19)
N1—C1—C6—C5	−177.46 (19)	C13—C12—C17—C8	179.96 (19)
C2—C1—C6—C5	0.9 (3)	C11—C12—C17—C8	−1.0 (3)
C1—N1—C7—C8	175.18 (19)	C7—C8—C17—C16	−3.2 (3)
N1—C7—C8—C9	−1.9 (3)	C9—C8—C17—C16	179.21 (19)
N1—C7—C8—C17	−179.45 (19)	C7—C8—C17—C12	177.07 (19)
C7—C8—C9—O1	4.3 (3)	C9—C8—C17—C12	−0.5 (3)

### Hydrogen-bond geometry (Å, °)

**Table:**

*D*—H···*A*	*D*—H	H···*A*	*D*···*A*	*D*—H···*A*
N1—H1N1···O1	1.01 (3)	1.61 (3)	2.505 (2)	146 (3)
N1—H1N1···N2	1.01 (3)	2.39 (3)	2.737 (3)	100 (2)
N2—H1N2···O1^i^	0.89 (3)	2.47 (3)	3.219 (3)	142.0 (19)
N2—H2N2···O1^ii^	0.95 (3)	1.98 (3)	2.879 (3)	158.6 (19)
C6—H6A···O3^iii^	0.93	2.57	3.489 (3)	168
C15—H15A···O2^iv^	0.93	2.51	3.161 (3)	127

Symmetry codes:  (i) −*x*+2, −*y*, −*z*+2; (ii) −*x*+2, −*y*−1, −*z*+2; (iii) −*x*+2, *y*+1/2, −*z*+3/2; (iv) −*x*+2, *y*+3/2, −*z*+3/2.
